# Data of vegetation structure metrics retrieved from airborne laser scanning surveys for European demonstration sites

**DOI:** 10.1016/j.dib.2025.111548

**Published:** 2025-04-09

**Authors:** W. Daniel Kissling, Wessel Mulder, Jinhu Wang, Yifang Shi

**Affiliations:** University of Amsterdam, Institute for Biodiversity and Ecosystem Dynamics (IBED), P.O. Box 94240, 1090 GE Amsterdam, the Netherlands

**Keywords:** Canopy height, Ecological remote sensing, Ecosystem structural complexity, Essential biodiversity variable, Habitat condition monitoring, Horizontal heterogeneity, Vegetation indices, Vertical profile

## Abstract

This dataset provides a standardized collection of rasterized Light Detection And Ranging (LiDAR) metrics in GeoTIFF format, derived from country-wide airborne laser scanning (ALS) data across seven demonstration sites in five European countries: Mols Bjerge National Park (Denmark), Reserve Naturelle Nationale du Bagnas (France), Oostvaardersplassen (Netherlands), Salisbury Plain (United Kingdom), Knepp Estate (United Kingdom), Monks Wood (United Kingdom), and the island of Comino (Malta). The sites range in areal size from 0.08 km^2^ to 54 km^2^ and include habitat types such as forests, broadleaf and conifer woodlands, small plantations, dry and wet grasslands, marshes, reedbeds, arable fields, farmland, scrublands and mediterranean garigue. A total of 35 LiDAR metrics were calculated, of which 28 represent vegetation structural attributes. These include vegetation height (seven metrics), vegetation cover (fourteen metrics), and vegetation vertical variability (seven metrics). Additionally, seven metrics describe point density (one metric), eigenvalues (three metrics), and normal vectors (three metrics). The rasterized LiDAR metrics have a spatial resolution of 10 m, with coverage and extent defined by shapefiles corresponding to each demonstration site. The raw ALS point clouds were clipped to the site boundaries and processed with the 'Laserfarm' workflow, a standardized computational workflow that includes modular pipelines for re-tiling, normalization, feature extraction, and rasterization. Laserfarm employs the feature extraction module of the open-source ‘Laserchicken’ software to compute the LiDAR metrics. The workflow was implemented using the IT services of the Dutch national facility for information and communication technology, SURF. The clipped LiDAR point clouds are available through a public repository, except for the LiDAR point clouds from Comino, Malta, which are not publicly available. The 35 rasterized LiDAR metrics (GeoTIFF files, 10 m resolution) from all sites, including Comino, as well as the corresponding site boundary shapefiles (geospatial vector format), are provided in a Zenodo repository. Additionally, the Jupyter Notebooks with Python code for executing the Laserfarm workflow are available to facilitate reproducibility and further computational applications. Users should note that the rasterized LiDAR metrics may contain zero or NA values, particularly over water surfaces, with the pulse penetration ratio metric potentially indicating false high vegetation cover over water. Users may reclassify or mask areas with zero values accordingly. Some pixels exhibit abnormal vegetation height values, which can be filtered before analysis. Certain striping patterns, likely resulting from overlapping flight lines and increased point density, are present in some metrics, though their overall impact appears minimal. This dataset enables diverse applications, including canopy height measurements, mapping of hedgerows, treelines, and forest patches, as well as characterizing vegetation density, vertical stratification, and habitat openness. It supports landscape-scale habitat analysis and contributes to the standardization of vegetation metrics from ALS data for site-specific ecological monitoring (e.g., Natura 2000). Moreover, the dataset demonstrates the automated execution of LiDAR data processing workflows, which is crucial for establishing a transnational and multi-site biodiversity and ecosystem observation network.

Specifications Table

**Table:** 

Subject	Earth & Environmental Sciences
Specific subject area	Quantifying and mapping vegetation structure with data from national airborne laser scanning surveys.
Type of data	Image (rasterized LiDAR metrics in GeoTIFF format) Shapefile (boundaries of demonstration sites in geospatial vector data format) Python code (Jupyter Notebooks)
Data collection	Raw point clouds from airborne laser scanning (ALS) surveys were clipped to the boundaries of seven demonstration sites. A computational workflow (called 'Laserfarm') with modular pipelines for re-tiling, normalization, feature extraction and rasterization was then applied to extract point cloud information from the ALS data. A total of 35 LiDAR metrics was calculated. Twenty-eight of these metrics reflect aspects of vegetation height, vegetation cover and vegetation vertical variability, and seven additional features reflect point density, three eigenvalues and three normal vectors. The LiDAR metrics were processed into raster layers (GeoTIFF files) with a 10 m resolution.
Data source location	Seven sites (ranging in size from 0.08 km^2^ to 54 km^2^) in five European countries (Denmark, France, Netherlands, United Kingdom, Malta) were included. These represent the demonstration sites from the Modern Approaches to the Monitoring of BiOdiversity (MAMBO) project (https://www.mambo-project.eu/), funded by the European Commission. The geographical coordinates of the approximate location (X, Y) of each demonstration site are as follows: • Mols Bjerge National Park (Denmark): 10.478393 E, 56.289050 N • Reserve Naturelle Nationale du Bagnas (France): 3.514360 E, 43.314332 N • Oostvaardersplassen (The Netherlands): 5.418842 E, 52.456870 N • Salisbury Plain (United Kingdom): 1.866189 W, 51.220814 N • Knepp Estate (United Kingdom): 0.3758 W, 50.969859 N • Monks Wood (United Kingdom): 0.2386 W, 52.400 N • Comino (Malta): 36.0113 E, 14.3362 N The input data source was public raw data of airborne laser scanning (ALS) point clouds from national repositories in Denmark, France, The Netherlands, and the United Kingdom. • Denmark: https://dataforsyningen.dk/data/3931 • France: https://geoservices.ign.fr/lidarhd#telechargement • The Netherlands: https://www.arcgis.com/home/webscene/viewer.html?layers=77da2e9eeea8427aab2ac83b79097b1a • United Kingdom: https://environment.data.gov.uk/DefraDataDownload/?Mode=survey For Malta, input data of ALS point clouds were not publicly accessible. They were provided by the Malta Planning Authority through Ecostack Innovations (https://www.ecostackinnovations.com/), an environmental consultancy firm. From the input data sources (national ALS point clouds), only tiles which contained the boundaries of the demonstration sites were included. This corresponded to the following tile indices: • Mols Bjerge National Park (Denmark): PUNKTSKY_623_59_TIF_UTM32-ETRS89 (6231_598, 6232_598, 6232_597, 6232_596, 6233_597, 6233_598) • Reserve Naturelle Nationale du Bagnas (France): LIDARHD_1-0_LAZ_MQ-0742_6247-2021, LIDARHD_1-0_LAZ_MQ-0740_6247-2021, LIDARHD_1-0_LAZ_MQ-0744_6247-2021, LIDARHD_1-0_LAZ_MQ-0742_6245-2021, LIDARHD_1-0_LAZ_MQ-0740_6245-2021, LIDARHD_1-0_LAZ_MQ-0740_6249-2021 • Oostvaardersplassen (The Netherlands): 26AN2, 26AZ2, 26BN1, 26BZ1, 20DZ2, 26BN2, 26BZ2 • Salisbury Plain (United Kingdom): SU0040_P_11780 • Knepp Estate (United Kingdom): TQ1020_P_10749 • Monks Wood (United Kingdom): TL2075_P_10756 • Comino (Malta): 438_3985, 438_3986, 439_3984, 439_3985, 439_3986, 440_3984, 440_3985, 440_3986, 441_3985, 441_3986 • Those tiles with LiDAR point clouds were then clipped using the boundary polygons (shapefiles) of the seven demonstration sites. Both the clipped LiDAR point clouds and the shapefiles of the MAMBO demonstration sites are provided in a publicly accessible repository (https://doi.org/10.48546/workflowhub.datafile.5.1), except for the LiDAR point clouds from Comino, Malta, which are not publicly available. The LiDAR metrics (GeoTIFF files with 10 m spatial resolution) from all demonstration sites (including Comino, Malta) together with the site boundaries (shapefiles in geospatial vector data format) are provided in a Zenodo repository (see below section ‘Data accessibility’).
Data accessibility	All data (i.e. 35 raster layers in GeoTIFF format and site boundaries in shapefile format for each demonstration site) are made publicly available [1]. Repository name: Zenodo Data identification number: DOI 10.5281/zenodo.14745309 Direct URL to data: https://doi.org/10.5281/zenodo.14745309 Instructions for accessing these data: Open access with CC BY 4.0 license
Related research article	None.

## 1 Value of the Data

• Airborne laser scanning (ALS) surveys provide high-resolution, three-dimensional representations of objects and features on the earth surface. This does not only include man-made structures (e.g. buildings, bridges, powerlines, vehicles, and archaeological sites) and terrain, but also natural features such as vegetation [2,3], including trees, shrubs, herbs and grasses. Processing these Light Detection and Ranging (LiDAR) point clouds into vegetation structure metrics can offer precise data on canopy height, vegetation cover and density, and vertical variability of plant biomass [[4], [5], [6]]. Vegetation structure metrics thus allow researchers to analyse habitat heterogeneity, biodiversity patterns, forest structure and ecosystem functions at fine spatial scales, enabling detailed ecological assessments in protected areas, nature reserves, agricultural landscapes and forest stands. ALS data therefore support forest inventories, ecosystem modelling and landscape-scale habitat connectivity analysis.
• ALS-derived vegetation structure metrics can serve as robust baseline datasets for tracking changes in vegetation dynamics over time [7,8]. They can provide information on tree heights, understory density, hedges, tree lines, plant cover, habitat openness, foliage height diversity, and horizontal vegetation heterogeneity [9]. Researchers can use these data to map habitats and land cover types [10], to analyse habitat preferences of animal species in relation to surface roughness, vegetation openness and woodland edge extent [11], or to monitor above-ground biomass and forest dynamics, e.g. in relation to the number and size of crowns and gaps [6]. It also allows researchers to detect disturbances, monitor succession processes, and assess the effectiveness of conservation measures in study sites such as Natura 2000 areas or other designated conservation zones [9]. These applications are valuable for conservation planning, land-use management, and ecosystem service evaluations, contributing to evidence-based decision-making in protected and managed landscapes.
• Rasterization of vegetation structure metrics makes the extracted features available as raster layers in data formats (e.g. GeoTIFF files) that are compatible with Geographic Information System (GIS) or other software familiar to ecologists (e.g. R). This facilitates a broad application of vegetation structure data because it avoids the time-consuming and often challenging process of storing, handling and processing big data volumes of ALS point clouds, which can be difficult for ecologists and other domain scientists [12,13]. The rasterized data can therefore be easily integrated with other ecological, climatological, and remote sensing data, supporting applications such as biodiversity and species distribution modelling, carbon stock estimation, ecological indicator assessments and habitat suitability analyses. This enhances interdisciplinary research opportunities by providing spatially explicit vegetation structural information that is applicable to diverse scientific fields.
• Applying standardised and robust pipelines for converting ALS point clouds into vegetation structure metrics promotes reproducibility and comparability of metric calculations and reusability of the generated data [6,14]. ALS-derived vegetation structure metrics can then be applied by researchers conducting comparative studies across different sites, ecosystems or geographic regions. The generation of transnational and multi-site datasets facilitates not only meta-analyses and synthesis studies but also supports the testing of ALS metric robustness in terms of different LiDAR point cloud characteristics (e.g. varying point or pulse densities and different point classifications, [6,9]) and choices of workflow parameter settings (e.g. for re-tiling, filtering, and specifying the coordinate reference system, [14]). These experiences are needed for developing a transnational biodiversity and ecosystem observation network and will help to identify broader ecological patterns and inform conservation and management strategies across multiple sites, datasets and countries.

## 2 Background

The LiDAR metrics were generated in the context of the Modern Approaches to the Monitoring of BiOdiversity (MAMBO) project [15] to demonstrate the feasibility of applying the Laserfarm workflow [14] to process airborne laser scanning (ALS) data from different European countries. The standardised approach allows to calculate metrics of vegetation height, cover and vertical variability, using LiDAR point clouds with different characteristics, such as varying point densities (5–15 pts/m^2^) and different standard point classes from the American Society for Photogrammetry & Remote Sensing (ASPRS). The transnational location and variation in areal coverage (0.08–54 km^2^) of the sites required to (1) access different national ALS repositories in Europe, (2) clip the LiDAR point clouds to the study area boundaries, (3) define site-specific re-tiling parameters such as X and Y coordinates of the bounding boxes and tile numbers (1–20 tiles per site), and (4) specify the coordinate reference system for each site using the standard from the European Petroleum Survey Group (EPSG). The work contributes to generating standardised vegetation metrics from airborne LiDAR data for site-specific (e.g. Natura 2000) monitoring, and to automate the execution of workflows for vegetation metric retrieval from ALS surveys.

## 3 Data Description

The Zenodo repository [1] contains a ZIP folder for each MAMBO demonstration site:

• **Bagnas.zip** (= Reserve Naturelle Nationale du Bagnas, France)
• **Comino.zip** (= Comino, Malta)
• **Knepp.zip** (= Knepp Estate, United Kingdom)
• **MolsBjerge.zip** (= Mols Bjerge National Park, Denmark)
• **MonksWood.zip** (= Monks Wood, United Kingdom)
• **Oostvaardersplassen.zip** (= Oostvaardersplassen, The Netherlands)
• **SalisburyPlain.zip** (= Salisbury Plain, United Kingdom)

Each ZIP folder provides (1) six Jupyter Notebooks (for running the Laserfarm workflow to extract the LiDAR metrics), plus two *.txt files with parameters settings (one with the EPSGS code of each country, one containing the grid details of the retiling schema), (2) the 35 LiDAR metrics in raster format with a 10 m resolution, provided as GeoTIFF files in two folders: ‘export’ (containing the raw tif files exported from Laserfarm) and ‘masked’ (the files masked using a boundary shapefile for each demonstration site), and (3) a visualization of each metric (i.e. a plotted map in PDF format). An additional PDF is provided which combines the maps of all LiDAR metrics per site in a single file.

The data of vegetation structure metrics described in this data paper are the 35 LiDAR metrics in raster format with a 10 m resolution (‘rasterized LiDAR metrics’). Besides the seven ZIP folders for the MAMBO demonstration site, the repository [1] contains the following additional files:

• **Shapefiles_StudySites.zip** (= seven ESRI shapefiles with the boundaries of the seven demonstration sites)
• **Methodology_description.pdf** (= a document containing a detailed description of the applied methodology)
• **README.txt** (= a Readme file describing the content of the repository)

### 3.1 Rasterized LiDAR metrics

**Fig. 1:**
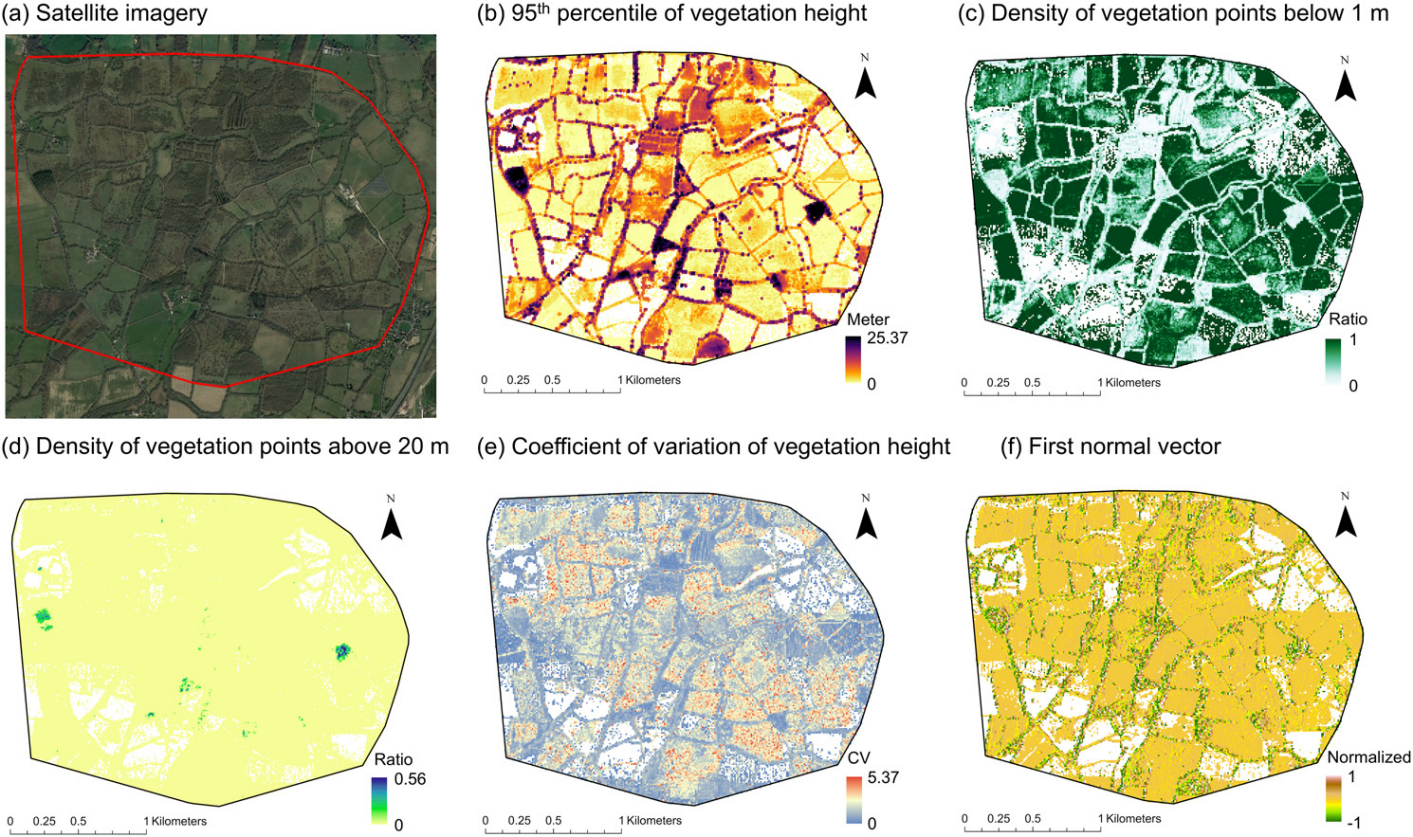
The demonstration site Knepp Estate in the United Kingdom (0.3758 W, 50.969859 N), with examples of rasterized LiDAR metrics at 10 m spatial resolution. (a) Satellite imagery from Google Earth Pro with the boundaries of Knepp Estate (red polygon). The site is an agricultural area and was once intensively farmed but is now devoted to rewilding and regenerative farming. (b) The 95th percentile of vegetation height as a measure of canopy height, capturing hedges, treelines and forest patches. (c) Density of vegetation points below 1 m, representing the proportion of vertical vegetation distribution in the lowest vegetation layer. (d) Density of vegetation points above 20 m as an indicator of the tallest vegetation patches in the landscape. (e) Coefficient of variation of vegetation height as a measure of vertical variability in plant biomass. (f) The first normal vector as a measure of the direction of the vegetation surface, capturing linear elements (hedges and tree lines) and planar surfaces (agricultural fields).

Thirty-five rasterized LiDAR metrics were calculated (Table 1). Twenty-eight out of the thirty-five metrics represent measures of vegetation structure, including metrics of vegetation height (seven metrics), vegetation cover (fourteen metrics) and vegetation vertical variability (seven metrics). Seven additional features reflect point density (one metric), eigenvalues (three metrics) and normal vectors (three metrics). Examples of the rasterized LiDAR metrics for one of the demonstration sites are illustrated in Fig. 1. The spatial resolution of the rasterized LiDAR metrics is 10 m, and the spatial extent and coverage is defined by the shapefile of each demonstration site. National ALS data (LiDAR point clouds) were used as input (see above ’Data source location’) and metric calculations were performed with the Laserfarm workflow [14] (see below ‘Experimental design, materials and methods’). The Laserfarm workflow uses the feature extraction module from the free-and-open-source ‘Laserchicken’ software [12] for the LiDAR metric calculations. The formal description of each feature calculation is provided on the Laserchicken's documentation website (https://laserchicken.readthedocs.io/en/latest/#features).

**Table 1:** The 35 LiDAR metrics extracted with the Laserfarm workflow for the seven MAMBO demonstration sites. The processed 10 m resolution GeoTIFF files for each demonstration site are available from the Zenodo repository [1].

Laserfarm feature	LiDAR metric name	Description
*Vegetation height*		
perc_25_normalized_height	25th percentile of vegetation height	25th percentile of normalized z within a 10 m × 10 m grid cell
perc_50_normalized_height	50th percentile of vegetation height	50th percentile of normalized z within a 10 m × 10 m grid cell
perc_75_normalized_height	75th percentile of vegetation height	75th percentile of normalized z within a 10 m × 10 m grid cell
perc_95_normalized_height	95th percentile of vegetation height	95th percentile of normalized z within a 10 m × 10 m grid cell
max_normalized_height	Maximum vegetation height	Maximum of normalized z within a 10 m × 10 m grid cell
median_normalized_height	Median of vegetation height	Median of normalized z within a 10 m × 10 m grid cell
mean_normalized_height	Mean of vegetation height	Mean of normalized z within a grid cell
*Vegetation cover*		
pulse_penetration_ratio	Pulse penetration ratio	Ratio of number of ground points (*N_ground_*) to the total number of points (*N_total_*) within a 10 m × 10 m grid cell
density_absolute_mean_normalized_height	Density of upper vegetation layer	Number of returns above mean height within a 10 m × 10 m grid cell
band_ratio_normalized_height_1	Density of vegetation points below 1 m	Ratio of number of vegetation points (< 1 m) to the total number of vegetation points within a 10 m × 10 m grid cell
band_ratio_1_normalized_height_2	Density of vegetation points between 1 and 2 m	Ratio of number of vegetation points (between 1 and 2 m) to the total number of vegetation points within a 10 m × 10 m grid cell
band_ratio_2_normalized_height_3	Density of vegetation points between 2 and 3 m	Ratio of number of vegetation points (between 2 and 3 m) to the total number of vegetation points within a 10 m × 10 m grid cell
band_ratio_normalized_height_3	Density of vegetation points below 3 m	Ratio of number of vegetation points (< 3 m) to the total number of vegetation points within a 10 m × 10 m grid cell
band_ratio_3_normalized_height	Density of vegetation points above 3 m	Ratio of number of vegetation points (> 3 m) to the total number of vegetation points within a 10 m × 10 m grid cell
band_ratio_3_normalized_height_4	Density of vegetation points between 3 and 4 m	Ratio of number of vegetation points (between 3 and 4 m) to the total number of vegetation points within a 10 m × 10 m grid cell
band_ratio_4_normalized_height_5	Density of vegetation points between 4 and 5 m	Ratio of number of vegetation points (between 4 and 5 m) to the total number of vegetation points within a 10 m × 10 m grid cell
band_ratio_normalized_height_5	Density of vegetation points below 5 m	Ratio of number of vegetation points (< 5 m) to the total number of vegetation points within a 10 m × 10 m grid cell
band_ratio_5_normalized_height_6	Density of vegetation points between 5 and 6 m	Ratio of number of vegetation points (between 5 and 6 m) to the total number of vegetation points within a 10 m × 10 m grid cell
band_ratio_5_normalized_height_20	Density of vegetation points between 5 and 20 m	Ratio of number of vegetation points (between 5 and 20 m) to the total number of vegetation points within a 10 m × 10 m grid cell
band_ratio_6_normalized_height_10	Density of vegetation points between 6 and 10 m	Ratio of number of vegetation points (between 6 and 10 m) to the total number of vegetation points within a 10 m × 10 m grid cell
band_ratio_20_normalized_height	Density of vegetation points above 20 m	Ratio of number of vegetation points (> 20 m) to the total number of vegetation points within a 10 m × 10 m grid cell
		
*Vegetation vertical variability*		
coeff_var_normalized_height	Coefficient of variation of vegetation height	Coefficient of variation of normalized z within a 10 m × 10 m grid cell
entropy_normalized_height	Shannon index	The negative sum of the proportion of points within 0.5 m height layers multiplied with the logarithm of the proportion of points within 0.5 m height layers within a 10 m × 10 m grid cell
kurto_normalized_height	Kurtosis of vegetation height	Kurtosis of normalized z within a 10 m × 10 m grid cell
sigma_z	Roughness of vegetation	Standard deviation of the residuals of a fitted plane within a 10 m × 10 m grid cell
skew_normalized_height	Skewness of vegetation height	Skewness of normalized z within a 10 m × 10 m grid cell
std_normalized_height	Standard deviation of vegetation height	Standard deviation of normalized z within a 10 m × 10 m grid cell
var_normalized_height	Variance of vegetation height	Variance of normalized z within a 10 m × 10 m grid cell
		
*Other features*		
point_density	Point density	Point density within a 10 m × 10 m grid cell
eigenv_1	First eigenvalue	First principal direction of variation within neighbourhoods around target points
eigenv_2	Second eigenvalue	Second principal direction of variation within neighbourhoods around target points
eigenv_3	Third eigenvalue	Third principal direction of variation within neighbourhoods around target points
normal_vector_1	First normal vector	First principal direction of the surface calculated from eigenvectors in three-dimensional space
normal_vector_2	Second normal vector	Second principal direction of the surface calculated from eigenvectors in three-dimensional space
normal_vector_3	Third normal vector	Third principal direction of the surface calculated from eigenvectors in three-dimensional space

All rasterized LiDAR metrics were calculated with the normalized point cloud. For vegetation structure metrics (except the pulse penetration ratio, Table 1), the Laserfarm workflow requires to specify which points are used as vegetation points. For all demonstration sites except the one in the Netherlands, the available ALS point clouds provided a pre-classification for vegetation points, using the ASPRS standard point classes from the LAS 1.4 format specification for low vegetation (class 3), medium vegetation (class 4), and high vegetation (class 5). For the ALS point cloud from the Netherlands, vegetation points are included in the ASPRS standard point class 'unclassified' (= 1). This class can introduce some biases in the calculation of vegetation metrics if a 10 m grid cell contains not only vegetation points, but also points from other objects such as cars, powerlines, transmission towers, boats, fences or poles [5,16]. However, this is negligible for the demonstration site Oostvaardersplassen in the Netherlands because such objects do not occur in this nature reserve, except for a few fences and poles (e.g. herbivore exclosures, fences around the demonstration site).

## 4 Experimental Design, Materials and Methods

### 4.1 Raw data

**Fig. 2:**
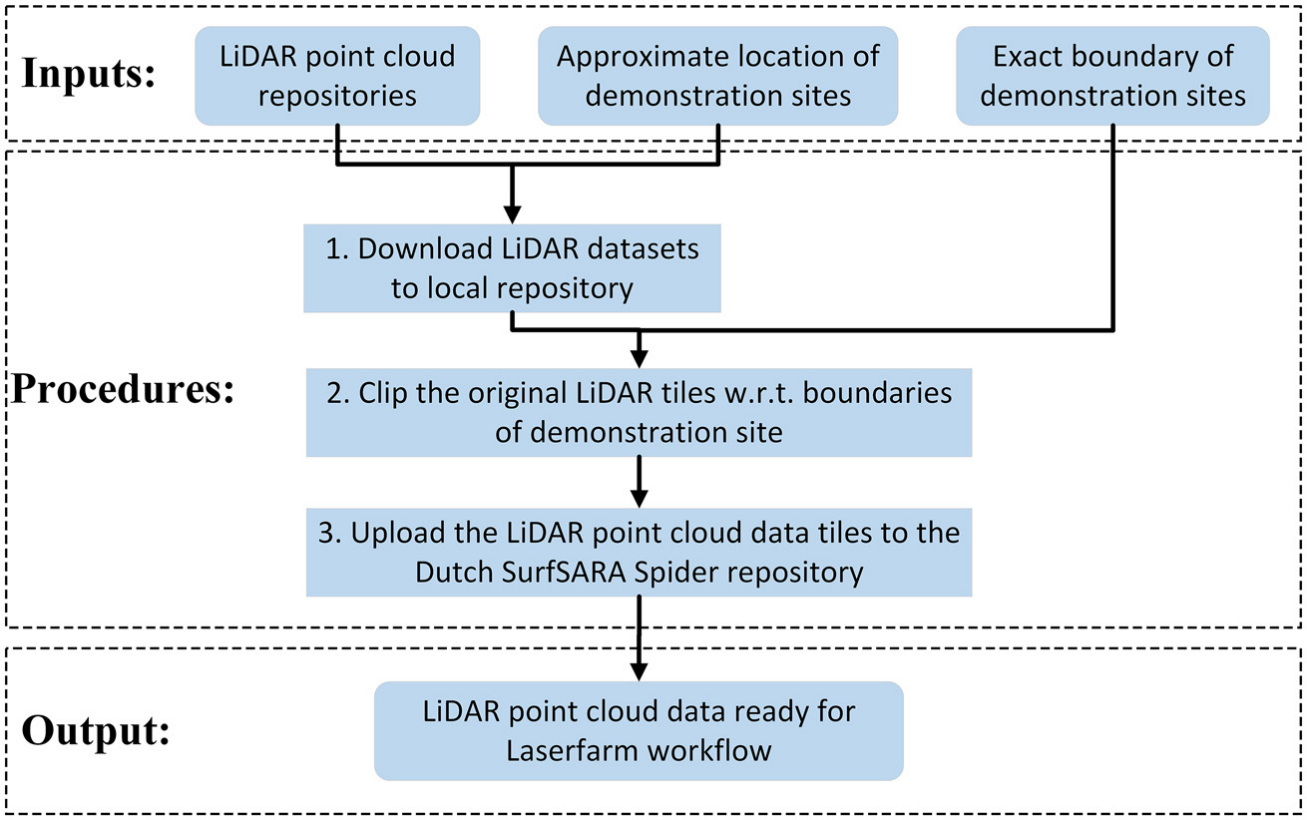
Pre-processing steps to prepare the raw LiDAR point clouds from different European countries for the subsequent calculation of rasterized LiDAR metrics with the Laserfarm workflow.

A few pre-processing steps were applied to prepare the raw LiDAR point clouds for each of the MAMBO demonstration sites (Fig. 2). These pre-processing steps required as input the raw LiDAR point clouds from the (national) repositories, the approximate location of the demonstration sites, and the ESRI shapefiles with the boundaries of the demonstration sites (Fig. 2).

In a first preprocessing step, the raw LiDAR point cloud tiles were downloaded from the national repositories in each country based on the approximate location of the demonstration sites (see approximate locations of sites and access links for raw datasets in Table 2). In a second step, the point cloud datasets were clipped with the boundary polygons (ESRI shapefiles) of the demonstration sites which are provided in the Zenodo repository [1]. In the third and final pre-processing step, the clipped LiDAR point clouds were uploaded to a data repository using the services from the Dutch IT infrastructure SURF (https://www.surf.nl/en). This repository is publicly accessible [17] and contains the six clipped LiDAR point clouds that are openly available (i.e. excluding the LiDAR point clouds from Comino, Malta, which are not openly available). The code for the pre-processing steps is available on GitHub (https://github.com/Jinhu-Wang/Retile_Clip_LAZ).

**Table 2:** Characteristics of LiDAR point clouds from the seven demonstration sites. Raw LiDAR point clouds were accessed from national airborne laser scanning datasets in five European countries and clipped to the boundaries of the demonstration sites using polygon shapefiles of the demonstration sites.

	Mols Bjerge National Park	Reserve Naturelle Nationale du Bagnas	Oostvaarders-plassen	Salisbury Plain	Knepp Estate	Monks Wood	Comino
Country	Denmark	France	Netherlands	United Kingdom	United Kingdom	United Kingdom	Malta
Site abbreviation	MolsBjerge	Bagnas	Oostvaardersplassen	SalisburyPlain	Knepp	MonksWood	Comino
Approximate location (x,y)	10.478393 E 56.289050 N	3.514360 E 43.314332 N	5.418842 E 52.456870 N	1.866189 W 51.220814 N	0.3758 W 50.969859 N	0.2386 W 52.400 N	36.0113 E 14.3362 N
Link to public raw data of LiDAR point clouds	https://dataforsyningen.dk/data/3931	https://geoservices.ign.fr/lidarhd#telechargement	https://www.arcgis.com/home/webscene/viewer.html?layers=77da2e9eeea8427aab2ac83b79097b1a	https://environment.data.gov.uk/DefraDataDownload/?Mode=survey	https://environment.data.gov.uk/DefraDataDownload/?Mode=survey	https://environment.data.gov.uk/DefraDataDownload/?Mode=survey	NA
Time of acquisition	2014–2015	2021–2022	2020 (AHN4)	2021	2019	2017, 2019	2018
Approximate areal coverage	1.29 km^2^	7.5 km^2^	54 km^2^	7.95 km^2^	5.55 km^2^	0.08 km^2^	3.5 km^2^
Indices of tiles	PUNKTSKY_623_59_TIF_UTM32-ETRS89 (6231_598, 6232_598, 6232_597, 6232_596, 6233_597, 6233_598)	LIDARHD_1–0_LAZ_MQ-0742_6247–2021, LIDARHD_1–0_LAZ_MQ-0740_6247–2021, LIDARHD_1–0_LAZ_MQ-0744_6247–2021, LIDARHD_1–0_LAZ_MQ-0742_6245–2021, LIDARHD_1–0_LAZ_MQ-0740_6245–2021, LIDARHD_1–0_LAZ_MQ-0740_6249–2021	26AN2, 26AZ2, 26BN1, 26BZ1, 20DZ2, 26BN2, 26BZ2	SU0040_P_11,780	TQ1020_P_10,749	TL2075_P_10,756	438_3985, 438_3986, 439_3984, 439_3985, 439_3986, 440_3984, 440_3985, 440_3986, 441_3985, 441_3986
Link to public location of clipped LiDAR point clouds	https://public.spider.surfsara.nl/project/lidarac/MAMBO/Data/DK/	https://public.spider.surfsara.nl/project/lidarac/MAMBO/Data/FR/	https://public.spider.surfsara.nl/project/lidarac/MAMBO/Data/NL/	https://public.spider.surfsara.nl/project/lidarac/MAMBO/Data/UK/	https://public.spider.surfsara.nl/project/lidarac/MAMBO/Data/UK/	https://public.spider.surfsara.nl/project/lidarac/MAMBO/Data/UK/	NA
Approximate average point density	10 pts/m^2^	10 pts/m^2^	15 pts/m^2^	7 pts/m^2^	7 pts/m^2^	7 pts/m^2^	5 pts/m^2^ (downsampled)
Local coordinate system	GEOGCS [Projection-""ERTS89"", Height-""DVR90""]	GEOGCS [Projection- ""WGS84"", Height - ""RGF93""]	GEOGCS [Projection- ""WGS84"", Height - ""RD_New""]	GEOGCS [Projection- ""WGS84""]	GEOGCS [Projection- ""WGS84""]	GEOGCS [Projection- ""WGS84""]	GEOGCS [Projection- ""WGS84""]
Point data record format	6	1	3	3	3	3	3
Classification available?	yes	yes	yes	yes	yes	yes	Yes
ASPRS standard point classes in demonstration site	ground (2); low vegetation (3); medium vegetation (4); high vegetation (5); building (6); low point - noise (7)	unclassified (1); ground (2); low vegetation (3); medium vegetation (4); high vegetation (5); water (9); powerline (64)	unclassified (1); terrain (2); buildings (6); water (9)	unclassified (1); ground (2); low vegetation (3); medium vegetation (4); high vegetation (5); buildings (6); low points - noise (7)	unclassified (1); ground (2); low vegetation (3); medium vegetation (4); high vegetation (5); buildings (6); low points - noise (7)	unclassified (1); ground (2); low vegetation (3); medium vegetation (4); high vegetation (5); buildings (6)	unclassified (1); ground (2); low vegetation (3); medium vegetation (4); high vegetation (5); buildings (6); water (9)
# returns	5	5	6	4	5	5	5
Other point cloud properties	Intensity, return number, scan angle rank, edge of flight lines, point source ID, GPS time	Intensity, return number, scan angle rank, edge of flight lines, point source ID, GPS time	Intensity, return number, scan angle rank, edge of flight lines, point source ID, GPS time	Intensity, return number, scan angle rank, edge of flight lines, point source ID, GPS time	Intensity, return number, scan angle rank, edge of flight lines, point source ID, GPS time	Intensity, return number, scan angle rank, edge of flight lines, point source ID, GPS time	Intensity, return number, scan angle rank, edge of flight lines, point source ID, GPS time

In addition to downloading, clipping and storing the raw LiDAR point clouds for each demonstration site, the properties of all raw data were examined and the summary information was extracted (details in Table 2). This was done using the CloudCompare software (https://www.danielgm.net/cc/). The LAS/LAZ files were loaded into the software and the relevant properties such as intensity, classification, GPS time, number of returns, edge of flight lines, point source ID, etc. were selected. To verify the specific property, the dataset was selected in the ‘DB tree’ panel of CloudCompare and the scalar field in the properties panel of the software was applied. The available values were then inspected in the active scalar fields and the characteristics of the raw LiDAR point clouds (e.g. time of acquisition, average point density, classification, number of returns and other point cloud properties) were extracted for each demonstration site (Table 2).

During point cloud inspection, it was noticed that one demonstration site (Mols Bjerge National Park) contained outliers from errors during the LiDAR data acquisition. Those outliers were removed using ‘Sparse Outlier Removal’ (SOR), a density-based filtering method [18]. This method detects sparse outliers by first computing the mean *µ* and standard deviation *σ* of the *κ* nearest neighbour distances. It then trims points that fall outside *µ*±*ασ*. The value of *α* depends on the size of the neighbourhood *κ*. The SOR tool in CloudCompare was used to perform the outlier cleaning. The point cloud data were first loaded and then selected in the ‘DB tree’ panel of CloudCompare. The ‘SOR’ button tool was then applied. The two parameters *α* and *κ* were set to 1 and 10, respectively.

### 4.2 Processing

The Laserfarm workflow (https://laserfarm.readthedocs.io/en/latest/) was applied to process the clipped LiDAR point clouds from each demonstration site into rasterized LiDAR metrics. Laserfarm is a high-throughput workflow for generating geospatial data products of vegetation structure using LiDAR point clouds from ALS surveys [14]. It is a free and open-source end-to-end workflow designed for the efficient, scalable and distributed processing of multi-terabyte LiDAR point clouds on remote infrastructures. The Laserfarm workflow is implemented in Python and builds on the free and open-source Laserchicken software [12] which has been specifically designed for extracting statistical properties of point cloud data from multi-terabyte datasets. Laserfarm contains four modular pipelines for (1) re-tiling, (2) normalization, (3) feature extraction and (4) rasterization [14]. These pipelines are available as Jupyter Notebooks [14]. The original Jupyter Notebooks of the Laserfarm workflow were developed during the processing of the country-wide LiDAR dataset from the third Dutch national ALS flight campaign [5]. These original Jupyter Notebooks were adapted and modified for implementing the Laserfarm workflow for the seven demonstration sites. A total of six Jupyter Notebooks were used for each demonstration site (Table 3). One Jupyter Notebook was used for re-tiling, one for normalization, and two Jupyter Notebooks for feature extraction and rasterization, respectively (Table 3). Two Jupyter Notebooks were needed here because the metric calculation required LiDAR point clouds with different input, i.e. one with vegetation points only (most metrics) and another one with all points (including ground points, only one metric). The code, i.e. all Laserfarm Jupyter Notebooks for each demonstration site, are published and documented in WorkflowHub (https://workflowhub.eu/projects/302#workflows), a registry for computational workflows. In addition, the Jupyter Notebooks are also provided in the Zenodo repository [1] of this data paper.

**Table 3:** Details of Jupyter Notebooks for implementing the Laserfarm workflow for the demonstration sites.

Jupyter Notebooks	Description
*Re-tiling*	
1_Retiling.ipynb	Re-tiles the original files from LiDAR repositories into smaller chunks for further efficient, scalable and distributed processing. Requires defining a grid-like structure for re-tiling and a spatial resolution (tile mesh size) for the final GeoTIFF files (here 10 m).
*Normalization*	
2_Normalization.ipynb	Normalizes the point cloud heights (z-values) relative to the terrain surface by calculating the normalized height for each individual point as the height relative to the lowest point within a grid cell. Requires defining a spatial resolution of the grid cell size for normalization (here 1 m).
*Feature extraction*	
3_Feature_extraction_veg.ipynb	Calculates LiDAR metrics (‘features’) with vegetation points, e.g. related to vegetation height, density, and vertical variability. Requires defining the spatial resolution (tile mesh size) for the metric calculation (here 10 m), the list of features, and the class(es) of points which are vegetation.
4_Feature_extraction_all.ipynb	Calculates LiDAR metrics (‘features’) of openness which use all points (not only vegetation points), namely the pulse penetration ratio (i.e. the ratio of the number of ground points to the total number of points within a grid cell). Requires defining the spatial resolution (tile mesh size) for the metric calculation (here 10 m) and the list of features (here only the pulse penetration ratio).
*Rasterization*	
5_Geotiff_export_veg.ipynb	Rasterizes the extracted features of vegetation (e.g. related to vegetation height, density, and vertical variability) and exports them as raster layers (here GeoTIFF format).
6_Geotiff_export_all.ipynb	Rasterizes the extracted features of openness (here pulse penetration ratio) and exports them as raster layers (here GeoTIFF format).

Implementing the Laserfarm workflow for the seven demonstration sites required a few user-defined parameter values (Table 4). The re-tiling Jupyter Notebook required to define a regular grid using the bounding box coordinates and the number of tiles along the side of the bounding box of each demonstration site (Table 4). The normalization Jupyter Notebook required to specify the spatial resolution for normalization which was defined to be a 1 m × 1 m grid cell (Table 4). The feature extraction Jupyter Notebooks required to define the set of LiDAR metrics (see the Laserfarm feature names in Table 1) and the ASPRS standard point classes for vegetation points (see Table 4). Finally, the rasterization Jupyter Notebooks required to specify the coordinate reference system using a standard machine-readable code from the European Petroleum Survey Group (EPSG) for coordinate systems worldwide (https://epsg.io/). Both the re-tiling Jupyter Notebook and the rasterization Jupyter Notebook had to access the specifications through text files (‘grids.txt’ and ‘epsgs.txt’, respectively) which are also provided in the Zenodo repository [1] of this data paper.

**Table 4:** User-defined parameter values for the Jupyter Notebooks implementing the Laserfarm workflow for the seven demonstration sites.

	Mols Bjerge National Park (Denmark)	Reserve Naturelle Nationale du Bagnas (France)	Oostvaarders-plassen (The Netherlands)	Salisbury plain (United Kingdom)	Knepp estate (United Kingdom)	Monks Wood (United Kingdom)	Comino (Malta)
*Re-tiling*							
min_x	596605.00	739243.00	144232.50	401539.00	512626.00	519397.00	438423.00
max_x	598945.00	744243.00	159232.50	403689.00	515626.00	520497.00	441823.00
min_y	6231377.00	6244168.00	488499.00	146344.00	118720.00	279015.00	3983780.00
max_y	6233717.00	6249168.00	503499.00	148494.00	121720.00	280115.00	3987180.00
n_tiles_side	2	20	15	1	3	1	1
*Normalization*							
normalize	1	1	1	1	1	1	1
*Feature extraction*							
features	See Appendix Table A4	See Appendix Table A4	See Appendix Table A4	See Appendix Table A4	See Appendix Table A4	See Appendix Table A4	See Appendix Table A4
apply_filter ‘value’ of ASPRS standard point classes for vegetation points	3,4,5	3,4,5	1	3,4,5	3,4,5	3,4,5	3,4,5
							
*Rasterization*							
current_epsg	25832	2154	28992	27700	27700	27700	32633

The IT services of the Dutch national facility for information and communication technology SURF (https://www.surf.nl/en/ict-facilities) were used to implement the Laserfarm workflow for the seven demonstration sites. SURF provides access to a national IT infrastructure for the Dutch academic community, including the versatile high-throughput data processing platform ‘Spider’ on which the computations were performed (https://www.surf.nl/en/services/high-performance-data-processing). Spider is an in house compute cluster of SURF which allows users to run highly parallel jobs on distributed resources, using scalable processing of many terabytes of data and utilizing many hundreds of cores simultaneously (https://servicedesk.surf.nl/wiki/display/WIKI/Spider). Several execution steps were conducted to set-up the Laserfarm Jupyter Notebooks on the Spider data processing platform. This included to make a directory, to build and activate the Jupyter-Dask environment, to install the Laserfarm software, to prepare the configuration files, and to start the Jupyter server (see details of execution steps in Table 5).

**Table 5:** Execution steps for setting up the Laserfarm workflow on the data processing platform ‘Spider’ of the Dutch IT infrastructure SURF (https://www.surf.nl/en/services/high-performance-data-processing). Shown are details of command-line interface and terminal executions that were necessary to set up the Jupyter Notebooks of the Laserfarm workflow.

Execution step	Command-line interface and terminal executions
Login to your compute cluster	*ssh <username>@hpc-environment.nl*
Make a directory for all files of interest	*mkdir PointClouds*
Build the Jupyter-Dask environment	*git clone*http://github.com/RS-DAT/JupyterDaskOnSLURM.git
Go to the directory	*cd PointClouds/JupyterDaskOnSLURM*
Create a new environment	*conda env create -f environment.yaml*
Activate the Jupyter-Dask environment	*conda activate jupyter_dask*
Install Laserfarm	*conda install pdal python-pdal gdal -c conda-forge; pip install Laserfarm*
Check and edit configuration files	*cat environment.yaml; vim config_spider.yml*
Copy the configuration file to home directory	*cp -r config/dask/config_spider.yml ∼/.config/dask/*
Go back to workspace	*cd /PointClouds/JupyterDaskOnSLURM*
Submit a job to start Jupyter server	*sbatch scripts/jupyter_dask_spider.bsh*
Check the job	*squeue -u <username>*
Copy the ssh command printed in the slurm-<JOB_ID>.out and paste it into a new terminal on your local machine. Access Jupyter session from your browser at localhost:8889	*ssh -i /path/to/private/ssh/key -N -L 8889:NODE:8888 <user>@hpc-environment.nl*.

## Limitations

**Fig. 3:**
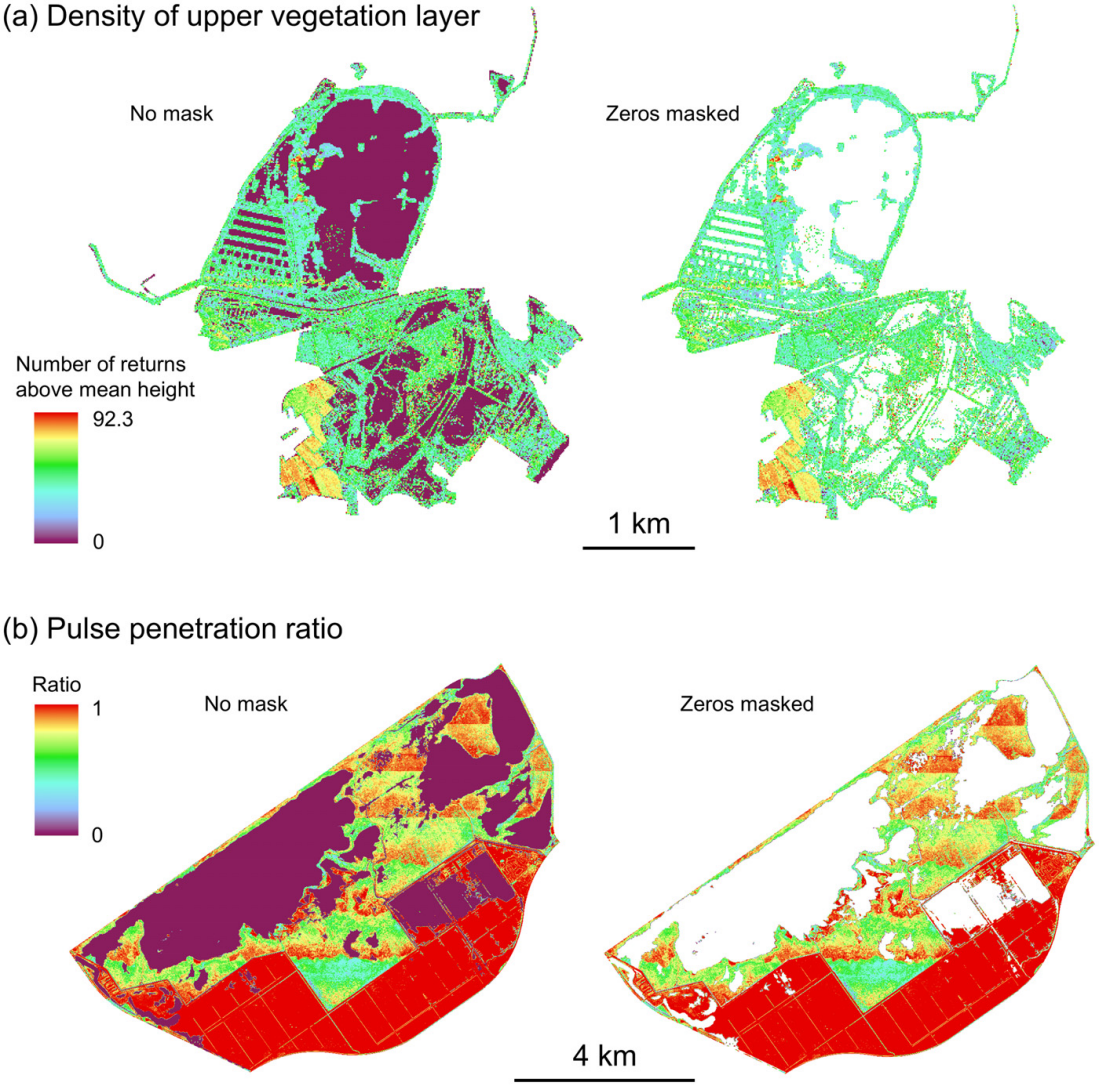
Examples of masking zero values in the rasterized LiDAR metrics. (a) The density of upper vegetation layer (‘density_absolute_mean_normalized_height’) in the Reserve Naturelle Nationale du Bagnas, France, has zero values in areas of lagoons (left, purple colour). They can be removed with masking (right). (b) The pulse penetration ratio ('pulse_penetration_ratio') in the Oostvaardersplassen of the Netherlands is a measure of openness (ratio of number of ground points to the total number of points) and can result in zero values (falsely indicating dense vegetation) in water areas of the marsh (left, purple colour). These can be removed by masking (right). Note that the large, red-coloured areas are grasslands that are intensively grazed by large herbivores (i.e. very open areas with little vegetation, hence a high pulse penetration ratio value).

For the interpretation of zero and NA values in the rasterized LiDAR metrics it is important to know that some Laserfarm features result in zero values if no vegetation points are present whereas other features result in NA values. For instance, ‘density_absolute_mean_normalized_height’, ‘entropy_normalized_height’, ‘point_density’, and some of the eigenvalues and normal vectors will result in zero values if no vegetation points are available in a grid cell. This happens, for instance, above water (if no vegetation is present), and water surfaces will then be represented by zeros (Fig. 3a left). However, other LiDAR metrics (i.e. all vegetation height metrics and all band ratios reflecting vegetation cover, Table 1) will result in NA values in such areas. Users could reclassify or mask areas with zero values for LiDAR metrics such as ‘density_absolute_mean_normalized_height’, ‘entropy_normalized_height’, ‘point_density’ etc. if they wanted to exclude such water areas for subsequent analyses (Fig. 3a right).

The pulse penetration ratio is the only rasterized LiDAR metric that uses not only vegetation points but all points. It is a measure of openness and calculated as the ratio of the number of ground points to the total number of points within a grid cell (Table 1). Above water, the pulse penetration ratio can result in zero values which falsely indicate a high vegetation cover (i.e. low openness, Fig. 3b left). This is caused by a lack of ground points above water surfaces which results in zero values when calculating the pulse penetration ratio. Users could either apply a water mask (e.g. if additional shapefiles or raster data reflecting water bodies are available for a specific study area), or mask areas with zero values of the pulse penetration ratio (Fig. 3b right).

**Fig. 4:**
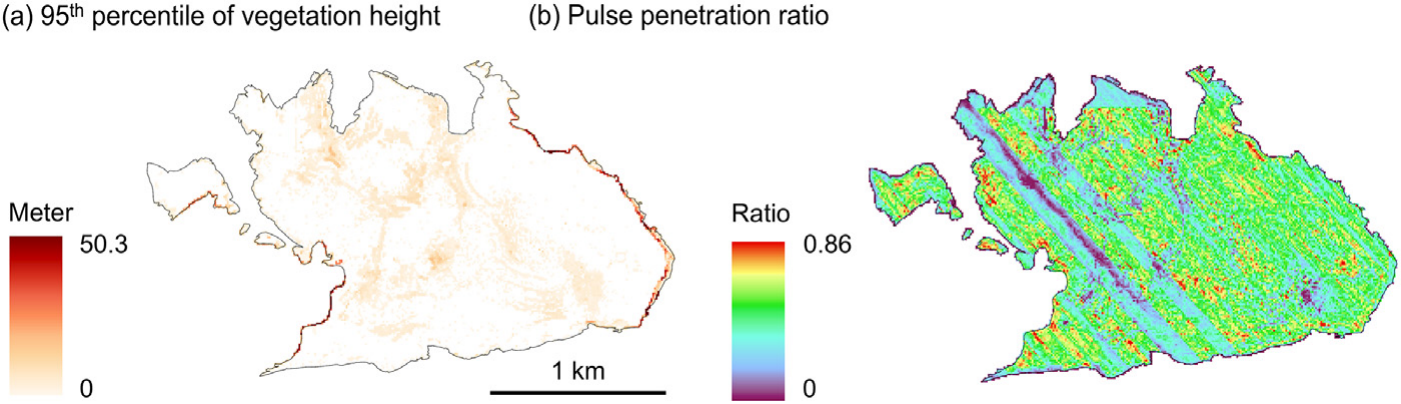
Specific issues observed in the rasterized LiDAR metrics of Comino, Malta. (a) Abnormal vegetation height values at the cliffs and edges of Comino. (b) Strip patterns from flightlines (NW to SE) evident in the pulse penetration ratio throughout the whole island.

A few pixels with abnormal vegetation height values exist in the rasterized LiDAR metrics. This is especially evident on the island of Comino, Malta, where steep terrain and limestone cliffs occur at the boundaries of the site (Fig. 4a). Those large vegetation height values (e.g. 50 m) are therefore most likely an artefact of the normalization method implemented in the Laserfarm workflow which may introduce inaccuracies in normalized vegetation height values, especially if steep terrain occurs at the scale of meters, i.e. within a 1 m × 1 m grid cell [14]. Most of the other demonstration sites have rather flat terrain, and vegetation height values above 30 m should be an exception. It is therefore recommended to filter out those abnormal values before using the rasterized LiDAR metrics for further analysis, e.g. by removing or masking grid cells with a 95th percentile of vegetation height > 30 m.

Some strip patterns occur in the rasterized LiDAR metrics. This is particularly evident in the pulse penetration ratio of Comino, Malta (Fig. 4b), and to a lesser extent in the pulse penetration ratio of the Oostvaardersplassen in the Netherlands (Fig. 3b). A possible reason could be the overlap of flight lines during data acquisition. Overlaps introduce multiple scanning angles, which enhance laser penetration through vegetation gaps by illuminating the same area from different directions. This increases point density unevenly and eventually can alter the pulse penetration ratio [19]. The strip issue is also visible in the point density feature of most sites but does not seem to strongly influence other LiDAR metrics that are calculated only with the vegetation points.

## Ethics statement

This work meets the requirements for ethical publishing (https://www.elsevier.com/authors/policies-and-guidelines). The work does not include chemicals, procedures or equipment that have any unusual hazards inherent in their use, nor does it involve the use of animal or human subjects, nor any data collected from social media platforms. No studies on patients or volunteers have been performed.

## Credit author statement

**W. Daniel Kissling:** Conceptualization, Formal analysis, Funding acquisition, Project administration, Supervision, Visualization, Writing –original draft, Writing –review & editing; **Wessel Mulder:** Formal analysis, Validation, Data Curation; **Jinhu Wang:** Formal analysis, Methodology, Software, Validation, Data Curation; **Yifang Shi:** Formal analysis, Methodology, Validation.

## Declaration of generative AI and AI-assisted technologies in the writing process

During the preparation of this work the authors used ChatGPT to create a preliminary version of the abstract and the section ‘Value of the data’. After using this tool, the authors re-wrote, refined and reviewed and edited the content and take full responsibility for the content of the publication.

## Data Availability

ZenodoExamples of applying the Laserfarm workflow to calculate LiDAR vegetation metrics across seven demonstration sites from five European countries (Original data).
